# Characterizing clinical pediatric obesity subtypes using electronic health record data

**DOI:** 10.1371/journal.pdig.0000073

**Published:** 2022-08-04

**Authors:** Elizabeth A. Campbell, Mitchell G. Maltenfort, Justine Shults, Christopher B. Forrest, Aaron J. Masino

**Affiliations:** 1 Department of Information Science, College of Computing & Informatics, Drexel University, Philadelphia, Pennsylvania, United States of America; 2 Department of Biomedical and Health Informatics, Children’s Hospital of Philadelphia, Philadelphia, Pennsylvania, United States of America; 3 University of Pennsylvania Perelman School of Medicine, Philadelphia, Pennsylvania, United States of America; 4 AiCure, New York, New York, United States of America; National Yang Ming Chiao Tung University, TAIWAN

## Abstract

In this work, we present a study of electronic health record (EHR) data that aims to identify pediatric obesity clinical subtypes. Specifically, we examine whether certain temporal condition patterns associated with childhood obesity incidence tend to cluster together to characterize subtypes of clinically similar patients. In a previous study, the sequence mining algorithm, SPADE was implemented on EHR data from a large retrospective cohort (n = 49 594 patients) to identify common condition trajectories surrounding pediatric obesity incidence. In this study, we used Latent Class Analysis (LCA) to identify potential subtypes formed by these temporal condition patterns. The demographic characteristics of patients in each subtype are also examined. An LCA model with 8 classes was developed that identified clinically similar patient subtypes. Patients in Class 1 had a high prevalence of respiratory and sleep disorders, patients in Class 2 had high rates of inflammatory skin conditions, patients in Class 3 had a high prevalence of seizure disorders, and patients in Class 4 had a high prevalence of Asthma. Patients in Class 5 lacked a clear characteristic morbidity pattern, and patients in Classes 6, 7, and 8 had a high prevalence of gastrointestinal issues, neurodevelopmental disorders, and physical symptoms respectively. Subjects generally had high membership probability for a single class (>70%), suggesting shared clinical characterization within the individual groups. We identified patient subtypes with temporal condition patterns that are significantly more common among obese pediatric patients using a Latent Class Analysis approach. Our findings may be used to characterize the prevalence of common conditions among newly obese pediatric patients and to identify pediatric obesity subtypes. The identified subtypes align with prior knowledge on comorbidities associated with childhood obesity, including gastro-intestinal, dermatologic, developmental, and sleep disorders, as well as asthma.

## Author summary

Childhood obesity is a significant public health challenge in the United States. Despite its prevalence, it remains uncertain if pediatric obesity represents a single condition or is composed of different subtypes with possibly different underlying causes. Electronic Health Records (EHRs) are an important source of data that may be analyzed to yield clinical and epidemiological insights to aid in the obesity treatment and prevention. In this paper, we present a study of EHR data that aimed to identify clinically similar subtypes among a population of newly obese pediatric patients. Specifically, we examine whether certain temporal condition patterns associated with childhood obesity incidence tend to cluster together to characterize subgroups of clinically similar patients. We identified eight potential subtypes, differentiated by the prevalence of various diagnoses including respiratory and sleep disorders, inflammatory skin conditions, asthma, and seizure disorders. This work may be used as a foundation for future investigations into pediatric obesity subtypes as well as to inform methodological and clinical research to mine EHR data for potential insights that improve patient health outcomes.

## Introduction

Approximately one third of children in the United States are overweight (age- and sex-specific body mass index (BMI) greater than or equal to the 85th percentile per Centers for Disease Control and Prevention (CDC) growth charts) or obese (age- and sex-specific BMI greater than or equal to the 95th percentile per CDC growth charts).[1,2] Obesity is linked with an increased risk of developing multiple comorbidities including asthma, diabetes, hypertension, and psychological conditions among pediatric patients during childhood and later in life.[3,4] Pediatric obesity is a socially significant health issue that disproportionately impacts American Indian, African American, and Latino children, compared to non-Hispanic whites.[5,6] Obesity prevalence is also higher among low-income, rural, or less-educated population subtypes.[5,7]

Despite its prevalence and social importance, it remains uncertain if childhood obesity represents a single condition or is composed of unique phenotypes with possibly different underlying causes. Grouping all types of overweight and obesity into one clinical condition may conceal associations between risk factors and specific subtypes of obesity, which has implications for improving prevention, recognition, and treatment of pediatric obesity.[8] Analyzing large healthcare datasets may potentially uncover previously unknown relationships concerning diagnoses, event patterns, and outcomes in healthcare, such as the presence of childhood obesity subtypes.

In recent years, use of such datasets in healthcare has increased.[9] Data sources include electronic health records (EHRs), medical imaging, wearable devices, genome sequencing, and payer records among others. Data mining methods for pattern discovery and extraction form a core set of methods to facilitate knowledge discovery from large healthcare datasets.[10,11] Data mining in healthcare may be used for numerous purposes including diagnostic outcomes evaluation, to uncover comorbidity and clinical event patterns, and to detect fraud and abuse.[12,13]

We present an investigation of EHR data to identify clinically similar subtypes among a population of newly obese pediatric patients. We examine whether certain temporal condition patterns associated with childhood obesity incidence tend to cluster together to characterize subtypes of clinically similar patients, and to describe the demographic characteristics of these patient subtypes. Specifically, we address the following:

1. Do temporal condition patterns associated with childhood obesity form clinically meaningful subtypes?
2. What are the demographic characteristics of patients within these subtypes?
3. How might associations in diagnostic clustering and demographic subtypes be used to advance clinical and public health pediatric obesity research?

## Materials and methods

Data from this study were derived from the Pediatric Big Data (PBD) resource at the Children’s Hospital of Philadelphia (CHOP) (a pediatric tertiary academic medical center). The PBD resource includes clinical data collected from CHOP, the CHOP Care Network (a primary care network of over 30 sites), and CHOP Specialty Care and Surgical Centers. Both clinical and non-clinical observations (as defined by Observational Health Data Sciences and Informatics (OHDSI) condition domain standards) from a patient’s EHR are included in the PBD database.[14] The PBD resource contains health-related information, including demographic, encounter, medication, procedure, and measurement (e.g. vital signs, laboratory results) elements for a large, unselected population of children. Non-study personnel extracted all data from the EHR and removed protected health information (PHI) identifiers, with the exception of dates, prior to transfer to the study database. Date information was removed from the analysis dataset as described below. The CHOP Institutional Review Board approved this study and waived the requirement for consent.

### Temporal condition patterns

In a previous study, [15] we applied a sequential pattern mining algorithm to a large retrospective cohort of patients (n = 49 694) from CHOP to identify common condition trajectories surrounding pediatric obesity incidence. This analysis used the CDC definition of childhood obesity (BMI z-score at or above the 95th percentile for age and sex).[16,17] Patients had at least one obesity measurement during a CHOP primary care visit and at least one visit prior to the first obesity measurement where an obese BMI was not recorded. The BMI z-scores were centrally calculated in this analysis. The same definition of obesity was used across study sites for the entire study period. Campbell, et al includes a full study diagram detailing the inclusion criteria implementation for obtaining the study population.[15]

EHR data from patients’ records for healthcare visits in which an obese BMI was first recorded (the index visit), as well as immediately before (pre-index visit) and after (post-index visit) were compiled for analysis. The presence of a pre-index visit was required for study inclusion to ensure that patients who became new patients in the CHOP healthcare system were not already obese. However, the presence of a post-index visit was not required for inclusion. Approximately two thirds of patients (67.6%) had a post-index visit.

The SPADE algorithm [18] was used to discover frequent temporal patterns among pre, index, and post visits in the study cohort. SPADE is a sequential pattern mining algorithm that finds frequent subsequence patterns from a larger sequence through an Apriori-based candidate generation method.[19] SPADE identified 163 condition patterns that were present in at least 1% of case patients. An example pattern is “1-ALL04, 2-EAR01” (a diagnosis of asthma in the pre-index visit, followed by a diagnosis of otitis media in the index visit). A control population of patients with a healthy BMI matched on age, prior healthcare visits, and sex was obtained and analyzed. We then examined prevalence in the control population of the common patterns identified among case patients. McNemar’s test results indicated that 80 of the 163 patterns were significantly more common among case patients (p<0.05). [15]

### Latent class analysis

The current study builds on results from Campbell, et al. and utilizes the same study population and temporal diagnoses that were previously identified. In this study, latent class analysis (LCA) [20] was used to identify potential subtypes formed by the diagnoses in temporal condition patterns that were significantly more common among obese pediatric patients. The assumption of conditional independence that underlies LCA is violated through the inclusion of both super-sequences and their frequent subsequences because each super-sequence is the intersection of its frequent subsequences. Therefore, only the frequent subsequences (i.e. individual temporal diagnoses) were included in our LCA modelling efforts. A total of 37 temporal diagnoses were evaluated to create patient subtypes in the LCA; these are listed in Table 1 in the Supplemental section. Each subsequence was considered as an individual feature in the dataset used for the LCA, with a binary value of 0 or 1 for if a patient had this temporal diagnosis or not.

**Table 1:** Latent Class Model Development Comparison.

Number of Classes	AIC	% reduction in AIC	BIC	% reduction in BIC
3	402 634	-	404 015	-
4	395 335	1.81%	397 179	1.69%
5	392 236	0.78%	394 543	0.66%
6	390 577	0.42%	393 347	0.30%
7	389 175	0.36%	392 408	0.24%
8	387 606	0.40%	391 302	0.28%

LCA requires specification of the number of classes as a user-selected parameter. Prior research on chronic diseases, including asthma, [21] diabetes, [22] and adult obesity [23] have indicated that there are typically 4–5 subtypes for these diseases. Input from clinician collaborators on this study suggested 8 classes as the maximum number that would be manageable and useful in care provision. Therefore, to obtain a clinically meaningful and interpretable number of patient subtypes, we elected to constrain our LCA evaluation to models with 3–8 classes.

The Akaike information criterion (AIC) and Bayesian information criterion (BIC) [24] were used to evaluate goodness-of-fit for each of the models tested.

R Version 3.6.1 was used for all data analysis in this study, [25] and the poLCA package [26] was used for the latent class modelling.

### Demographic subtype analysis

The LCA model assigns a probability of membership for each subtype (class) for a given individual. To facilitate analysis, using the final clustering model, each patient was assigned to the group for which he/she had the highest probability of membership. The high-prevalence diagnoses within each LCA-identified subtype, defined as those with ≥ 10% prevalence among patients, were used to clinically describe and name the subtypes. Finally, demographic information from patients’ EHR was incorporated to describe the patient subtypes. The demographic variables considered were sex, race, Medicaid enrollment (a proxy for socioeconomic status at the time of obesity incidence), [27,28] age at index visit (with age evaluated as both a continuous and categorical variable), and Philadelphia residence. Patients were classified as Hispanic if their self-identified ethnicity was specified as Hispanic or Latino; otherwise they were categorized by the value of their self-identified race the EHR. Patients with missing race and ethnicity information were classified as unknown.

If patients used multiple insurance types during their index visit, they were classified as being enrolled in Medicaid if one of those insurance plans was Medicaid or Children’s Health Insurance Program (CHIP), Pennsylvania’s state program to provide health insurance to uninsured children and teens who are ineligible or not enrolled in Medicaid.[29] If a patient did not have insurance information recorded for their index visit, all insurance information for patients’ visits within a year of their index visits was obtained from the PBD database and analyzed. If patients had a record of Medicaid/CHIP enrollment within a year of their index visit, then they were classified in the Medicaid/CHIP enrollment category (Medicaid/CHIP eligibility is assessed annually).[30] One hundred patients did not have any insurance information for a visit within a year of the index visit, and were dropped, leaving a total study population of 49,594 patients.

The frequency of categorical demographic variables (sex, race, Medicaid enrollment, age at index visit, and Philadelphia residence) and mean and standard deviation (SD) of continuous variables (mean age at index visit) were provided overall and for each subtype.

### Code availability

The code used for data processing and analysis in this study may be found at: https://github.com/chop-dbhi/masino-lab-obesity-incidence.

## Results

### Model selection

Table 1 presents the AIC and BIC values for each iteration, as well as the percent reduction in each criterion between models. As the number of classes in the latent class models increased, AIC and BIC values both declined. The model with 8 latent classes had both the lowest AIC and BIC values, and was the final model selected to study clinical subtypes among the study population.

### Clinical subtypes

Table 2 shows the prevalence rates for all 37 temporal diagnoses evaluated in the LCA among the 8 classes and the total study population. The high-prevalence diagnoses (≥ 10% prevalence) used to characterize the eight subtypes are highlighted.

**Table 2:** Prevalence Rate of Diagnoses by Class*.

Diagnostic Cluster	Class 1	Class 2	Class 3	Class 4	Class 5	Class 6	Class 7	Class 8
*Allergy*								
1-Asthma w/o Status Asthmaticus	4.58%	6.73%	3.00%	50.20%	4.86%	4.69%	2.96%	4.58%
2-Allergic Rhinitis	9.63%	11.89%	0.63%	31.06%	4.20%	3.47%	2.39%	7.15%
2-Asthma w/o Status Asthmaticus	6.08%	0.03%	2.84%	98.37%	0.27%	2.16%	3.53%	5.98%
*Ear Nose Throat*								
1-Deafness hearing loss	10.92%	0.99%	2.76%	2.09%	4.46%	2.82%	20.10%	0.17%
1-Chronic pharyngitis and tonsillitis	33.05%	0.29%	0.16%	0.93%	0.79%	0.56%	0.62%	0.07%
1-ENT Disorders other	7.53%	0.40%	0.87%	0.81%	1.32%	0.94%	2.29%	0.17%
2-Chronic pharyngitis and tonsillitis	45.51%	0.27%	0.16%	0.26%	0.00%	0.19%	0.10%	0.34%
3-Chronic pharyngitis and tonsillitis	33.99%	0.32%	0.39%	0.67%	0.06%	0.38%	0.62%	0.48%
*Gastrointestinal/Hepatic*								
1-Constipation	0.73%	1.82%	1.34%	1.63%	0.17%	47.07%	1.87%	1.81%
1-Gasteroenteritis	0.60%	1.84%	0.63%	0.73%	0.12%	1.97%	1.30%	22.56%
2-Constipation	1.63%	1.68%	1.58%	1.88%	0.00%	61.76%	4.31%	1.98%
2-Gastroesophageal reflux	3.17%	0.61%	3.55%	3.18%	1.15%	8.82%	4.21%	1.71%
*General Signs and Symptoms*								
1-Fever	1.28%	1.63%	2.29%	2.42%	0.24%	1.78%	0.31%	30.02%
1-Nausea vomiting	0.34%	0.00%	0.71%	0.42%	0.00%	1.88%	0.36%	20.00%
*Genito-Urinary*								
1-Urinary Symptoms	1.50%	1.34%	0.39%	1.74%	2.15%	18.58%	0.68%	0.51%
2-Urinary Symptoms	1.28%	1.66%	0.39%	1.16%	2.20%	17.60%	0.36%	1.71%
*Neurologic*								
1-Headaches	0.43%	0.11%	0.39%	1.09%	0.09%	0.23%	0.57%	28.10%
1-Seizure Disorder	0.47%	0.16%	62.32%	0.22%	0.10%	0.80%	0.57%	0.96%
1-Sleep Problems	21.79%	0.24%	0.87%	0.20%	0.23%	0.38%	1.77%	0.24%
1-Autism Spectrum Disorder	0.09%	0.11%	3.16%	0.20%	0.02%	0.23%	26.81%	0.03%
2-Neurologic signs and symptoms	1.84%	0.61%	10.19%	0.59%	1.54%	1.50%	11.12%	2.19%
2-Headaches	1.11%	0.96%	2.05%	0.76%	0.40%	0.28%	1.14%	21.81%
2-Seizure Disorder	0.68%	0.11%	79.15%	0.08%	0.03%	0.23%	1.35%	0.82%
2-Sleep Problems	22.35%	0.29%	0.95%	0.40%	0.31%	0.80%	5.09%	1.57%
2-Developmental disorder	3.42%	2.30%	17.69%	1.95%	2.37%	3.00%	49.82%	2.29%
2-Autism Spectrum Disorder	0.47%	0.05%	4.82%	0.61%	0.01%	0.84%	47.12%	0.38%
3-Seizure Disorder	0.47%	0.27%	46.84%	0.16%	0.07%	0.19%	0.78%	0.10%
3-Sleep Problems	17.81%	0.48%	0.55%	0.40%	0.07%	0.00%	2.08%	0.55%
3-Developmental disorder	2.31%	1.15%	10.82%	0.70%	1.15%	1.13%	36.21%	1.20%
3-Autism Spectrum Disorder	0.39%	0.21%	2.29%	0.09%	0.00%	0.47%	29.87%	0.27%
*Respiratory*								
1-Respiratory signs and symptoms	11.90%	2.35%	1.26%	4.72%	1.85%	1.50%	0.88%	1.33%
1-Sleep Apnea	23.54%	0.29%	1.18%	0.84%	0.43%	0.75%	0.42%	0.00%
2-Respiratory signs and symptoms	12.37%	4.25%	1.34%	4.33%	1.68%	1.36%	1.25%	2.80%
2-Sleep Apnea	26.50%	0.00%	0.79%	0.99%	0.03%	0.61%	0.36%	0.44%
3-Sleep Apnea	19.82%	0.16%	0.87%	0.40%	0.03%	0.33%	0.99%	0.55%
*Skin*								
1-Dermatitis and eczema	1.54%	54.21%	0.32%	9.28%	0.42%	1.41%	2.13%	0.58%
2- Dermatitis and eczema	2.40%	60.65%	1.66%	13.68%	0.08%	0.89%	2.86%	3.04%

* Highlighted prevalence rates are those greater than or equal to 10%.

The high-prevalence diagnoses for the LCA-derived classes were aggregated and evaluated to characterize each subtype (Table 3). Patients in Class 1 had a high prevalence of upper respiratory and sleep disorders, including sleep apnea and chronic pharyngitis and tonsillitis. Inflammatory skin conditions (i.e. dermatitis and eczema) were common among patients in Class 2. Seizures and other neurological disorders were prevalent among patients in Class 3, as was asthma among patients in Class 4. No condition pattern was prevalent at a rate at or above 5% among patients in Class 5; thus, patients in this subtype were characterized by their lack of a clear morbidity pattern (which also may include patients without any of the temporal condition patterns). Gastrointestinal and genitourinary symptoms were common among patients in

**Table 3:** Prevalence Rates of Common Temporal Diagnoses by Patient Subgroup (n = 49 594 patients).

Class (N)	Diagnoses
*Class 1 (n = 2 336)*: Upper Respiratory and Sleep Disorders	1-Chronic pharyngitis and tonsillitis (33.05%) 1-Deafness, hearing loss (10.92%) 1-Respiratory signs and symptoms (11.90%) 1-Sleep Apnea (23.54%) 1-Sleep Problems (21.79%) 2-Chronic pharyngitis and tonsillitis (45.51%) 2-Respiratory signs and symptoms (12.37%) 2-Sleep Apnea (26.5%) 2-Sleep Problems (22.35%) 3-Chronic pharyngitis and tonsillitis (33.99%) 3-Sleep Apnea (19.82%) 3-Sleep Problems (17.81%)
*Class 2 (n = 3 743)*: Inflammatory Skin Conditions	2-Allergic Rhinitis (11.89%) 1-Dermatitis and eczema (54.21%) 2-Dermatitis and eczema (60.65%)
*Class 3 (n = 1 266)*: Seizure Disorders and Epilepsy	1-Seizure Disorder (62.32%) 2-Developmental disorder (17.69%) 2-Neurologic signs and symptoms (10.19%) 2-Seizure Disorder (79.15%) 3-Seizure Disorder (46.84%) 3-Developmental disorder (10.82%)
*Class 4 (n = 6 446)*: Asthma	1-Asthma w/o Status Asthmaticus (50.20%) 2-Allergic Rhinitis (31.06%) 2-Asthma w/o Status Asthmaticus (98.37%) 2-Dermatitis and eczema (13.68%)
*Class 5 (n = 28 821)*: Other- no characteristic morbidity pattern	No diagnoses recorded at ≥ 5% prevalence
*Class 6 (n = 2 131)*: Gastrointestinal/Genitourinary Symptoms	1-Constipation (47.07%) 1-Urinary Symptoms (18.58%) 2-Constipation (61.76%) 2-Urinary Symptoms (17.60%)
*Class 7 (n = 1 925)*: Neurodevelopmental disorders	1-Autism Spectrum Disorder (26.81%) 1-Deafness, hearing loss (20.10%) 2-Autism Spectrum Disorder (47.12%) 2-Developmental disorder (49.82%) 2-Neurologic signs and symptoms (11.12%) 3-Autism Spectrum Disorder (29.87%) 3-Developmental disorder (36.21%)
*Class 8 (n = 2 925)*: Physical Symptoms	1-Fever (30.02%) 1-Gasteroenteritis (22.56%) 1-Headaches (28.10%) 1-Nausea, vomiting (20.00%) 2-Headaches (21.81%)

Class 6, and neurodevelopmental disorders (such as Autism) were prevalent among patients in Class 7. Finally, patients in Class 8 had a high prevalence of physical symptoms including headaches, fever, and nausea/vomiting.

The mean and standard deviation of the probability that patients categorized in their respective subtypes belong in that group, as well as their mean probability of membership in other classes is presented in Table 4. The mean probability of class membership to assigned groups ranged from 70.21% (patient in Class 8) to 89.7% (patients in Class 1). The mean probability of membership to other classes (those that patients in each group were not assigned to), ranged from 0.09% (the probability of patients in Class 3 belonging to Class 2 or Class 4) to 18.8% (patients in Class 5 belonging to Class 8).

**Table 4:** Mean Probability of Class Membership (Mean (SD))*.

Class	1	2	3	4	5	6	7	8
**1**	89.7% (18.02)	1.19% (3.38)	0.11% (1.22)	1.10% (4.76)	4.51% (9.68)	0.91% (3.08)	0.88% (3.56)	1.57% (4.08)
**2**	0.65% (3.16)	80.64% (13.78)	0.09% (1.30)	2.41% (4.79)	9.99% (9.40)	2.24% (5.56)	0.69% (2.58)	3.28% (5.47)
**3**	0.50% (2.76)	0.60% (1.91)	86.87% (19.83)	0.13% (1.13)	6.10% (11.68)	0.84% (2.43)	2.44% (6.04)	2.52% (5.65)
**4**	0.63% (3.10)	2.47% (5.01)	0.09% (1.38)	87.33% (14.9)	5.20% (7.87)	1.29% (4.33)	0.39% (2.31)	2.59% (5.15)
**5**	0.98% (3.43)	8.21% (6.26)	0.16% (1.12)	1.03% (2.71)	77.77% (11.86)	4.46% (5.84)	1.45% (3.70)	5.95% (4.65)
**6**	0.66% (3.03)	3.43% (4.69)	0.24% (2.09)	0.81% (3.44)	6.04% (9.42)	81.95% (15.74)	1.77% (5.09)	5.09% (7.84)
**7**	1.14% (4.54)	1.40% (3.96)	1.01% (3.74)	0.68% (3.75)	8.25% (13.05)	2.01% (5.01)	84.00% (18.7)	1.52% (3.72)
**8**	0.70% (3.29)	3.98% (5.09)	0.46% (3.14)	2.20% (7.45)	18.83% (16.17)	2.95% (5.45)	0.68% (2.67)	70.21% (19.5)

* The cell highlighted in grey represents the mean (SD) probability that patients belonged to their assigned class; orange cells represent probabilities above 5% that a patient belonged to a class different than their assigned one

### Demographic analysis

Demographic data obtained from patients’ EHRs were used to characterize the total study population (Table 5) and patient subtypes (Table 6). The majority of patients (55.3%) were male (n = 27 447). The racial composition was 47.0% White, 33.8% Black or African American, 8.8% Hispanic, and 2.3% Asian. At the time of the index visit, 41.2% of patients were enrolled in Medicaid and 37.2% were Philadelphia residents. Finally, 30.5% of patients were between two- and four-years-old, 42.9% were between five and eleven years, and 26.6% were between twelve and eighteen years. The mean age of patients at the time of the index visit was 8.5 years.

**Table 5:** Demographic Characteristics of Obesity Incidence Study Population.

	Study Population (n = 49 594)
	n (%)
Gender	
*Male*	27 447 (55.3%)
*Female*	22 147 (44.7%)
Race	
*Asian*	1 116 (2.3%)
*Black/African American*	16 741 (33.8%)
*White*	23 325 (47.0%)
*Hispanic*	4 350 (8.8%)
*Multiple Race*	466 (1.0%)
*Heterogeneous Other*	65 (<1%)
*Unknown*	3 511 (7.1%)
Medicaid Enrollment	
*Medicaid/CHIP*	20 439 (41.2%)
Age at index visit	
*2–4 years*	15 142 (30.5%)
*5–11 years*	21 255 (42.9%)
*12–18 years*	13 197 (26.6%)
Mean age at index visit (years)	
*Mean (SD)*	8.5 (4.6)
Philadelphia Residence	
	18 467 (37.2%)

**Table 6:** Demographic Characteristics of Obesity Incidence Study Population Clinical Subgroups.

	Upper Respiratory Disorders (n = 2 336)	Inflamm. Skin Conditions (n = 3 743)	Seizure Disorders and Epilepsy (n = 1 266)	Asthma (n = 6 446)	No Char. Morbidity Pattern (n = 28 821)	Gastro. Symptoms (n = 2 131)	Neuro dev. disorders (n = 1 925)	Physical Symptoms (n = 2 925)
	n (%)	n (%)	n (%)	n (%)	n (%)	n (%)	n (%)	n (%)
Sex								
*Male*	1291 (55.3%)	1899 (50.7%)	730 (57.7%)	3746 (58.1%)	15782 (54.8%)	1024 (48.1%)	1493 (77.6%)	1482 (50.7%)
*Female*	1045 (44.7%)	1844 (49.3%)	536 (42.3%)	2700 (41.9%)	13040 (45.2%)	1107 (51.9%)	432 (22.4%)	1443 (49.3%)
Race								
*Asian*	60 (2.6%)	109 (2.9%)	28 (2.2%)	120 (1.9%)	625 (2.2%)	47 (2.1%)	58 (3.0%)	69 (2.4%)
*Black/African American*	673 (28.8%)	1692 (45.2%)	366 (28.9%)	3372 (52.3%)	8332 (28.9%)	677 (31.8%)	417 (21.7%)	1212 (41.4%)
*White*	1105 (47.3%)	1342 (35.9%)	643 (50.8%)	2011 (31.2%)	14900 (51.7%)	1023 (48.0%)	1081 (56.2%)	1220 (41.7%)
*Hispanic*	256 (11.0%)	275 (7.3%)	154 (12.2%)	509 (7.9%)	2509 (8.7%)	209 (9.8%)	198 (10.3%)	240 (8.2%)
*Multiple Race*	30 (1.3%)	27 (<1%)	18 (1.4%)	55 (<1%)	284 (1.0%)	20 (<1%)	27 (1.4%)	25 (<1%)
*Heterogeneous Other*	3 (<1%)	6 (<1%)	2 (<1%)	3 (<1%)	68 (<1%)	4 (<1%)	4 (<1%)	2 (<1%)
*Unknown*	209 (8.9%)	292 (7.8%)	55 (4.3%)	376 (5.8%)	41 (7.4%)	151 (7.1%)	140 (7.3%)	157 (5.4%)
Medicaid Enrollment								
*Medicaid/CHIP*	987 (42.3%)	1746 (46.6%)	650 (51.3%)	3265 (50.7%)	10503 (36.4%)	883 (41.4%)	1100 (57.1%)	1305 (44.6%)
Age at index visit								
*2–4 years*	731 (31.3%)	1746 (46.6%)	488 (38.5%)	1927 (29.9%)	8156 (28.3%)	621 (29.1%)	820 (42.6%)	653 (22.3%)
*5–11 years*	1206 (51.6%)	1420 (37.9%)	474 (37.4%)	3143 (48.8%)	11860 (41.1%)	1065 (50.0%)	789 (41.0%)	1298 (44.4%)
*12–18 years*	399 (17.1%)	577 (15.4%)	304 (24.0%)	1376 (21.3%)	8806 (30.6%)	445 (20.9%)	316 (16.4%)	974 (33.3%)
Mean age at index visit (years)								
*Mean (SD)*	7.7 (4.0)	6.8 (4.3)	7.9 (4.8)	8.2 (4.3)	9.0 (4.7)	8.2 (4.2)	7.1 (4.3)	9.5 (4.7)
Philadelphia Residence								
	736 (31.5%)	1747 (46.7%)	396 (31.3%)	3471 (53.8%)	9354 (32.5%)	754 (35.3%)	585 (30.3%)	1424 (48.7%)

The demographic analysis of patients in the LCA-derived classes indicated that females tended to have higher rates of gastrointestinal issues; they comprised a majority of patients in Class 6. Males had a much higher prevalence of neurodevelopmental disorders. More than three quarters (77.6%) of patients in Class 7 were male. More than half of patients in Class 4 (Asthma) were African American (52.8%), despite African American patients comprising approximately one third of the total study population. African American patients comprised similarly high proportions of patients in Class 2 (45.2%) and Class 8 (41.4%) (Class 2 and Class 8 were characterized by having a high prevalence of inflammatory skin conditions and physical symptoms respectively). Class 2, Class 4, and Class 8 also had high proportions of urban youth compared to the total study population.

Medicaid enrollment was higher among patients in Class 3 (Seizures Disorders and Epilepsy), Class 4 (Asthma), and Class 7 (Neurodevelopmental Disorders) than among the total study population. Newly obese patients with neurodevelopmental disorders (Class 7) tended to be younger, suggesting that obesity may occur earlier among patients with Autism and developmental disorders. Finally, seizure disorders and epilepsy had higher prevalence among Hispanics; 12.2% of patients in Class 3 were Hispanic, compared to 8.8% of the total study population.

## Discussion

### Pediatric obesity subclasses

The preceding study utilized temporal condition patterns surrounding the time of obesity incidence that were previously identified as occurring at a significantly higher rate among obese pediatric patients compared to matched pediatric patients with a healthy BMI. These temporal condition patterns were used as input features to develop an LCA model with eight classes. Patients were assigned to the class for which they had the highest probability of membership. The mean probability of membership to the assigned class was high (>70% for each class) and the mean probability of belonging to a different class was low (<20%), suggesting a shared clinical characterization within the individual groups. The common condition patterns that occurred at a high prevalence rate (≥ 10% prevalence) among patients in each subtype were used to characterize the eight LCA-derived classes. Finally, the demographic characteristics of patients in each class were analyzed.

Our findings reflect extant literature on known comorbidities associated with pediatric obesity, including sleep issues as well as dermatologic, endocrine, gastro-intestinal, neurologic, musculoskeletal, and psychosocial conditions.[3,31] While our findings reflect current clinical knowledge on known pediatric obesity comorbidities, the presence of these diagnoses at obesity onset suggest there may be a bi-directional relationship between the conditions.

Additionally, there was a strong association between patients with both asthma and allergic rhinitis in Group 4. Prior research has shown a strong association between pediatric obesity and asthma development, as well as the possibility that early-life asthma contributes to pediatric obesity onset.[32–34] However, the relationship between allergic rhinitis is less clear. Some studies did not find a strong association between allergic rhinitis and obesity.[35,36] Han, et al.[37] found that centrally obese children had a reduced odds of developing allergic rhinitis but Lei, et al.[38] found that pediatric patients with overweight and obesity had an increased risk of developing allergic rhinitis. Our results indicate that comorbid asthma may mediate the relationship between allergic rhinitis and pediatric obesity, which may guide future research and clinical care provision aimed at preventing obesity among pediatric patients with allergic rhinitis.

From a demographic standpoint, similarities in the characteristics of our subtypes both align with and challenge prior clinical knowledge. As in our study, African American, low-income, and urban patients are known to be disproportionately represented among the asthma and inflammatory skin conditions subtypes.[39–41] Our study found a strong association between male sex and neurodevelopmental disorders (such as Autism Spectrum Disorder). While the link between autism and pediatric obesity is well established, [42] the strong link between sex, obesity, and neurodevelopmental disorders present in this study has not been seen in others.[43–45] Additionally, prior research has shown an equal distribution between sexes or male preponderance for gastro-intestinal conditions, [46–48] while in our study, a majority of patients in the Gastrointestinal/Genitourinary Symptoms subclass were female. This suggests that obesity may influence the association between sex and gastrointestinal disorder prevalence.

### Strengths and limitations

This study presents a data-driven approach to uncover pediatric obesity subtypes from a large electronic health record dataset. The study used a high volume of data from a randomly sampled population, which strengthens its goals to identify possible obesity subtypes without assuming an a priori hypothesis. Additionally, while LCA allows individuals to have a probability of membership in multiple subtypes, our results showed subjects generally had a very high membership probability for a single class (>70%). This suggests shared clinical characterization within the individual groups. However, it is important to note our study’s limitations. When working with such a large number of temporal condition patterns, certain constraints must be considered. Given the potential for false discovery rate, multiple comparisons testing would be necessary if attempting to establish statistical significance of the associations discovered using this approach. A further limitation comes from the somewhat arbitrary limits imposed in our study definitions (namely the 10% prevalence threshold for “high prevalence” conditions and limiting the number of LCA classes to a maximum of 8 in the modelling efforts). It is possible that a larger number of classes may achieve a better AIC or BIC score; however, one of our objectives was to constrain the number of classes to be clinically manageable.

### Future work

Our LCA modelling results suggest the existence of obesity subtypes. Obese patients with similar comorbidities at the time of obesity incidence may have similar future health trajectories. The clinical subtypes identified in our study can serve as hypothesis generation for such patient classes, whose future health outcomes can be explored. This would allow more specialized clinical care for obese pediatric patients with certain comorbidities.

Obesity is a complex and socially significant health issue that may affect different clinical and demographic subtypes of pediatric patients differently. Our findings can support the work of public health researchers and practitioners who seek to address the social disparities component of the obesity epidemic. By better understanding the needs and population demographics of obese pediatric patients, and the comorbidities that certain newly obese patients may be more likely to develop, the epidemiology of pediatric obesity can be better studied and resources can be better allocated to populations in need.

Future research may utilize the identified subtypes as hypotheses for possible pediatric obesity subtype to explore causality in the associations uncovered in this study. Understanding both the demographic characteristics and physical comorbidities that differentiate obese pediatric patients can help to better understand the etiology of the condition and appropriate treatment for the diverse groups of patients the condition affects. Additionally, this work shows the possibility for future researchers to utilize temporal condition patterns to develop classification models that could identify the clinical subtype for individuals newly diagnosed with pediatric obesity. Developing data-driven subtypes using diagnostic information in patient EHR data represents an exciting new frontier for researchers across clinical domains.

## Supporting information

S1 Table Statistically Significant Temporal Diagnoses among Newly Obese Pediatric Patients (n = 49 594 patients). The numbers before each diagnosis in a sequence represents the diagnosis timing class: ‘1’ denotes that the observation was recorded during a patient’s pre-index visit, ‘2’ represents the index visit, and ‘3’ signifies the post-index visit.
(DOCX)
