# Supplementary material for: Characterizing clinical pediatric obesity subtypes using electronic health record data
Source: PLOS Digit Health. 2022 Aug 4;1(8):e0000073. doi: 10.1371/journal.pdig.0000073 (PMC9931247; doi:10.1371/journal.pdig.0000073)
Supplement: S1 Table — The numbers before each diagnosis in a sequence represents the diagnosis timing class: ‘1’ denotes that the observation was recorded during a patient’s pre-index visit, ‘2’ represents the index visit, and ‘3’ signifies the post-index visit. (DOCX) [file pdig.0000073.s001.docx]

**S1 Table.** Statistically Significant Temporal Diagnoses among Newly Obese Pediatric Patients (n= 49 594 patients). The numbers before each diagnosis in a sequence represents the diagnosis timing class: ‘1’ denotes that the observation was recorded during a patient’s pre-index visit, ‘2’ represents the index visit, and ‘3’ signifies the post-index visit.

| *Allergy* |
| --- |
| 1-Asthma w/o Status Asthmaticus |
| 2-Allergic Rhinitis |
| 2-Asthma w/o Status Asthmaticus |
| *Ear, Nose, Throat* |
| 1-Deafness, hearing loss |
| 1-Chronic pharyngitis and tonsillitis |
| 1-ENT Disorders, other |
| 2-Chronic pharyngitis and tonsillitis |
| 3-Chronic pharyngitis and tonsillitis |
| *Gastrointestinal/ Hepatic* |
| 1-Constipation |
| 1-Gasteroenteritis |
| 2-Constipation |
| 2-Gastroesophageal reflux |
| *General Signs and Symptoms* |
| 1-Fever |
| 1-Nausea, vomiting |
| *Genito-Urinary* |
| 1-Urinary Symptoms |
| 2-Urinary Symptoms |
| *Neurologic* |
| 1-Headaches |
| 1-Seizure Disorder |
| 1-Sleep Problems |
| 1-Autism Spectrum Disorder |
| 2-Neurologic signs and symptoms |
| 2-Headaches |
| 2-Seizure Disorder |
| 2-Sleep Problems |
| 2-Developmental disorder |
| 2-Autism Spectrum Disorder |
| 3-Seizure Disorder |
| 3-Sleep Problems |
| 3-Developmental disorder |
| 3-Autism Spectrum Disorder |
| *Respiratory* |
| 1-Respiratory signs and symptoms |
| 1-Sleep Apnea |
| 2-Respiratory signs and symptoms |
| 2-Sleep Apnea |
| 3-Sleep Apnea |
| *Skin* |
| 1-Dermatitis and eczema |
| 2- Dermatitis and eczema |
